# Upright Perception and Ocular Torsion Change Independently during Head Tilt

**DOI:** 10.3389/fnhum.2016.00573

**Published:** 2016-11-17

**Authors:** Jorge Otero-Millan, Amir Kheradmand

**Affiliations:** ^1^Department of Neurology, The Johns Hopkins University School of MedicineBaltimore, MD, USA; ^2^Department of Otolaryngology-Head and Neck Surgery, The Johns Hopkins University School of MedicineBaltimore, MD, USA

**Keywords:** subjective visual vertical, SVV, upright perception, torsional eye position, ocular torsion, aftereffect, head tilt

## Abstract

We maintain a stable perception of the visual world despite continuous movements of our eyes, head and body. Perception of upright is a key aspect of such orientation constancy. Here we investigated whether changes in upright perception during sustained head tilt were related to simultaneous changes in torsional position of the eyes. We used a subjective visual vertical (SVV) task, modified to track changes in upright perception over time, and a custom video method to measure ocular torsion simultaneously. We tested 12 subjects in upright position, during prolonged (~15 min) lateral head tilts of 20 degrees, and also after the head returned to upright position. While the head was tilted, SVV drifted in the same direction as the head tilt (left tilt: −5.4 ± 1.4° and right tilt: +2.2 ± 2.1°). After the head returned to upright position, there was an SVV aftereffect with respect to the pre-tilt baseline, which was also in the same direction as the head tilt (left tilt: −3.9 ± 0.6° and right tilt: +2.55 ± 1.0°). Neither the SVV drift nor the SVV aftereffect were correlated with the changes in ocular torsion. Using the Bayesian spatial-perception model we show that the pattern of SVV drift and aftereffect in our results could be explained by a drift and an adaptation in sensory inputs that encode head orientation. The fact that ocular torsion (mainly driven by the otoliths) could not account for the perceptual changes suggests that neck proprioception could be the primary source of drift in upright perception during head tilt, and subsequently the aftereffect in upright position.

## Introduction

We maintain a stable perception of the visual world despite continuous movements of our eyes, head and body. A key aspect of such “orientation constancy” is the internal estimate of the direction of gravity that is used by the brain to compensate for the changes in the head and body positions. As a result, the visual scene is perceived as upright despite its changing orientation on the retina. This internal estimate of upright is often studied by measuring a perceived orientation of a visual line in an otherwise dark room, referred to as the subjective visual vertical (SVV; Howard, 1982; Van Beuzekom and Van Gisbergen, 2000).

To estimate upright orientation during the SVV task, the brain must integrate information about the visual line on the retina with other sensory inputs that encode head, eye and body orientations. In upright position, the vertical meridians of the eye, head and body are all aligned with the gravity axis, and SVV remains accurate usually within 2° of the earth vertical (Howard, 1982; Van Beuzekom and Van Gisbergen, 2000). With a lateral head tilt towards the shoulder, there is a compensatory torsional eye movement known as the ocular counterroll, which is usually far less than the amount of head tilt (5–25%). Therefore, during a lateral head tilt, the vertical meridian of the eyes no longer aligns with the axis of gravity and the images do tilt on the retina (Collewijn et al., 1985; Groen et al., 1999; Bockisch and Haslwanter, 2001). Now, to estimate upright orientation, the brain has to integrate information about the head and body positions in space and the eye position in head. This complex process leads to systematic errors and lower precision of SVV during head tilt than in upright position. With a large head tilt (beyond 60°) the SVV error is typically in the direction of the head tilt (known as Aubert or A effect), whereas with a smaller head tilt (below 60°) the SVV error may be in the direction opposite to the head tilt (known as Müller or E effect; Howard, 1982; Van Beuzekom and Van Gisbergen, 2000).

Perception of upright and torsional position of the eyes may not remain steady during a static head tilt (Wade, 1970; Pansell et al., 2005; Tarnutzer et al., 2012, 2013). The pattern of drift in SVV responses could be variable across individual subjects (Tarnutzer et al., 2013). SVV often drifts in the direction of the head tilt and also shows a post-tilt bias referred to as the aftereffect (Wade, 1968, 1970; Tarnutzer et al., 2012, 2013, 2014; Kheradmand et al., 2015). Ocular torsion may also drift, usually in the direction of the head tilt (Diamond and Markham, 1983; Pansell et al., 2005). Such drifts have important implications for understating the mechanisms behind orientation constancy with changes in head or eye position.

Here we measured SVV and ocular torsion simultaneously to address whether the drift in upright perception during head tilt was related to torsional eye position. We used a modified SVV paradigm to track changes in upright perception over time. While it is technically challenging to measure torsional eye position, we have developed a novel video method that allows tracking ocular torsion in real time (Otero-Millan et al., 2015). First, we examined the correlation between changes in SVV and ocular torsion during head tilt and their corresponding aftereffects when the head was brought back to upright position. Second, we examined the correlation between the drift and aftereffect separately for SVV and ocular torsion. Finally, we used a Bayesian spatial-perception model to simulate our findings and discuss a possible mechanism for the drift in upright perception during head tilt (De Vrijer et al., 2009).

## Materials and Methods

### Experimental Setup

**Figure 1:**
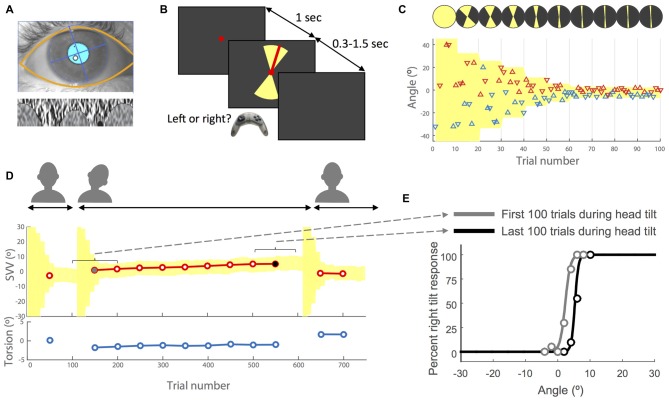
**Experimental methods. (A)** Example of eye image obtained from the cameras with the overlay of automatically detected eyelids and pupil (top) and the iris pattern extracted and optimized to calculate torsion (bottom). For more details see Otero-Millan et al. (2015). **(B)** Subjective visual vertical (SVV) paradigm. In each trial, subjects fixated on a red dot for 1 s before the line appeared. They had 1.5 s to respond whether the line was tilted to the left or to the right of what they perceived as upright (two alternative forced choice or 2AFC). This was done by pressing the left or right button on a controller. The line was presented within a range of possible angles (yellow shade) that varied during the experiment (see insets **C,D**). After pressing the button, the line disappeared and the next trial started with a new line orientation. **(C)** Sample time course of the first 100 trials with the subject’s responses. Each triangle represents one trial. The *y*-axis shows the angle of the line presented and the color indicates the subject’s response for that trial. The left tilt responses are shown in blue and the right tilt responses in red. The triangles that point up indicate the line was presented in the upper hemifield and the triangles that point down indicate the line was presented in the lower hemifield. The line angles were presented randomly within a range that started at 360° and then adjusted based on previous responses (illustrated by the top circles with yellow sectors). At the end of every 10 trials, the center of this range (yellow shade) was set as the SVV value calculated from previous 30 trials. The amplitude of the range was also adjusted every 10 trials by dividing it in half until it reached 10° (±5° around the calculated center), after which it was kept constant for the rest of the trials. **(D)** Sample recording of torsional eye position (bottom) and SVV (top) during an entire session. Each SVV point is calculated from a psychometric fit to the responses from 100 trials (see inset **E**) and each torsion point corresponds with the average ocular torsion during the same block of 100 trials. First, the head is in the upright position, then tilted and finally back again in the upright position. Every time the head changed position from upright to tilt or tilt to upright, the paradigm reset and angles were presented starting again at the full range of 360° (yellow shade). **(E)** Examples of psychometric fits corresponding with the first block of 100 trials (gray dot in **D**) and the last block of 100 trials during the head tilt (black dot in **D**). The SVV for each block is calculated as the center of the psychometric curve (50% right and left responses).

The experiments were approved by the Johns Hopkins institutional review board and informed consent was obtained from all the participants. Twelve healthy volunteers (mean age 29 years, 9 females) participated in this study. SVV and ocular torsion were recorded simultaneously in a completely dark room. The head was immobilized using a molded bite bar while the visual stimulus appeared on a CRT monitor (1280 pixel by 1024 pixel) 135 cm away from the subject. We mounted the bite bar on a rotary motor (Zaber Technologies Inc., Vancouver, BC, Canada) in order to change the head tilt position remotely. Each subject participated in two experiment sessions, one with the head tilted 20° to the right, and one with the head tilted 20° to the left, in a random order across subjects. We chose to tilt the head on the body as it has been previously shown that it can produce a relatively large SVV tilt and aftereffect (Wade, 1968). In each session, the subject stayed on the bite bar for the whole time. The SVV was first recorded in upright position, and after 100 trials the bite bar was tilted remotely to record 500 trials while the head remained in the static tilt position. The bite bar was then tilted back to upright to record 150 more trials (total of 750 trials). We added a 30-s pause in the SVV paradigm after each time the head changed position (Figure 1D), in order to avoid residual effects from the semicircular canal stimulation during head movement.

### Ocular Torsion

We used RealEyes xDVR system manufactured by Micromedical Technologies Inc., Chatham, IL, USA and a custom software to record torsional eye position. This system uses two cameras (Firefly MV, PointGrey Research Inc., Richmond, BC, Canada) mounted on goggles to capture infrared images of each eye. To measure and track torsional eye position, we used a method developed by our group (Figure 1A) that operates binocularly in real time at 100 Hz and with a noise level less than 0.1° (Otero-Millan et al., 2015).

### Adaptive SVV Paradigm

The software that controlled the SVV paradigm was written in Matlab (Mathworks) using Psychtoolbox (Kleiner et al., 2007). A red line (length: 7.6° of visual angle, width: 0.13°) was presented in random angles around a fixed, red dot at the eye level (diameter: 0.33°; Figure 1B). The perceived line orientation was measured using a two alternative forced choice (2AFC) task. In each trial, subjects clicked the right or left button on a controller to report whether the line was tilted to the right or left of what they perceived as upright. The line orientation was randomly selected within a range that was adjusted in blocks of 10 trials (Figures 1B,C). In each block, five different angle orientations were presented in the top of the visual field (always radiating from the fixation point) and equivalent five angles were presented in the bottom of the visual field. In each trial, the fixation dot appeared first, and then after 1 s, the visual line was presented for a minimum of 300 ms and maximum of 1.5 s until subject responded. If the subject did not respond within 1.5 s, the line disappeared and a new trial started after a button click. In such cases, the angle that was missed was presented again at a later time within the same block to ensure that all angles were presented exactly once. At the beginning of the paradigm, the angles were selected randomly from the entire range of 360°, but as the paradigm continued the range of probing angles was adjusted frequently to precisely track temporal changes in subject’s perception. To make this adjustment, every 10 trials a new range was calculated that: (1) was centered around the SVV calculated from the responses in previous 30 trials (for SVV calculation see “Data Analysis” Section); and (2) had an amplitude that decreased by half until the paradigm reached the 9th block, after which it remained constant at 10° for the rest of the trials (Figures 1C,D). Thereby, the SVV paradigm could adapt, track changes in subject’s perception, and it was not biased by making any prior assumption about the SVV value. Every time the head changed position from upright to tilt or tilt to upright, the paradigm reset to the starting range of 360° (Figure 1D).

### Data Analysis

SVV was calculated by fitting a psychometric curve to the responses using a logistic function and a generalized linear regression model (Matlab fitglm). The SVV value was the angle at which the probability of left or right responses was 50% (point of subjective equality). The SVV precision was calculated as the difference between the 50% and 75% points on the psychometric curve.

In order to compare ocular torsion and SVV responses, we first calculated the average torsional position of the two eyes during each trial in the SVV paradigm. Then within a window of 100 trials, we calculated the average ocular torsion and fitted a psychometric curve to the responses from these 100 trials to calculate the SVV value (Figure 1E). The window then advanced in steps of 50 trials to obtain more SVV and ocular torsion values. The first 50 trials were discarded as the range of angles in these initial trials was not narrow enough to get a reliable SVV value (Figure 1D). We used a simple linear regression to measure the drift over time and estimate the rate of change for both SVV and ocular torsion. To calculate correlations across subjects, we first averaged the values for the right and left head tilts and then used Spearman method to obtain the coefficient. For comparisons, we used *t*-test with a significant *p*-value less than 0.01.

## Results

**Figure 2:**
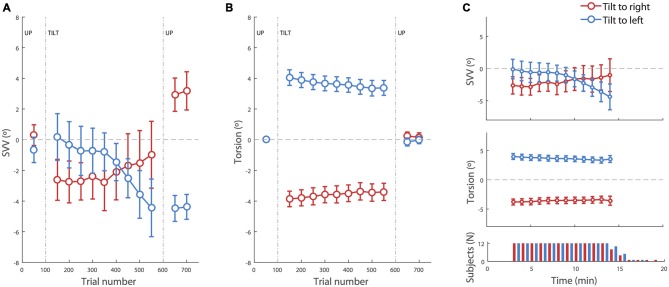
**SVV and torsional position of the eyes during prolonged head tilts. (A)** Average SVV during head tilts to the right (red) and left (blue). Each point corresponds with the SVV calculated from responses within 100 trials. The gaps in the data correspond with the first 50 trials in the new head position where SVV estimates were not reliable and were discarded. **(B)** Average torsional eye position during head tilts to the right (red) and left (blue). As in the SVV plot, each point corresponds with the average ocular torsion within the blocks of 100 trials, and the gap in the data corresponds with the first 50 trials that were discarded. **(C)** Top and middle: same data as in **(A,B)** during head tilt aligned in time (instead of trial numbers) show the same pattern of drift in SVV and ocular torsion. The averages are shown for the time points where the SVV data was available from more than half of the subjects. Bottom panel: number of subjects from which data was recorded at each time point during the right and left head tilts. Subjects took different amounts of time to complete the 500 trials. In all panels, the error bars correspond with SEM across subjects.

We used an adaptive psychophysical paradigm to track temporal changes in perception of upright during head tilt, while simultaneously recording ocular torsion. Subjects started in upright position and after 100 trials the head was passively rolled 20° to the left or right. Then after 500 more trials (~15 min) the head was brought back to upright position. Figure 2 shows the averages of SVV reports and ocular torsion during the experiment. The initial recording in the upright position served as the baseline for torsional position of the eyes. Thus, all subsequent torsion measurements were relative to this baseline value.

There was an average SVV of −0.2 ± 0.6° at the baseline upright position, combining both head tilt positions. Separating left and right head tilt positions, the average SVV responses during the baseline upright position were −0.7° and 0.2° respectively, and not significantly different (*t*-test, *p* = 0.4). At the beginning of the head tilt, subjects showed an initial bias in SVV responses that was measured during the first 100 trials. In this initial tilt period, more subjects were biased away from the head tilt (i.e., showed an E effect) than towards the head tilt (i.e., showed an A effect), consistent with previous reports of E-effect for a small head tilt (20° in our case; Van Beuzekom and Van Gisbergen, 2000). The average SVV at the beginning of the left head tilt was +0.2 ± 1.5° and the average SVV at the beginning of the right head tilt was −2.6 ± 1.3°. This asymmetry was however not significant (*t*-test *p* = 0.2). In addition, the initial SVV value did not predict the amount of SVV drift during head tilt (*r* = 0.2, *p* = 0.3). The initial torsional position of the eyes with the left and right head tilts were respectively +4.1 ± 0.5° and −3.8 ± 0.5° (which corresponds with gains of +20 ± 2% and −19 ± 2% respectively). Unlike the SVV values, the ocular torsion for all subjects was in the opposite direction of the head tilt (i.e., the ocular counterroll). There was no significant asymmetry between the ocular torsion for the right and left head tilts (*t*-test *p* = 0.7).

Neither SVV nor ocular torsion remained stable during head tilt. SVV drifted towards the direction of the head tilt in 10 of 12 subjects. That is, when the head was tilted to the right, SVV drifted towards the right, and when the head was tilted to the left, the SVV drifted towards the left. By approximating the drift as a linear function, an average drift was determined as the slope of the linear fit to the data from all subjects. The group average for the SVV drift during 500 trials (~15 min) was −5.4 ± 1.4° for the left head tilt and +2.2 ± 2.1° for the right head tilt. The drift was symmetrical, i.e., there was no difference between the values for the right tilt and the reversed values for the left tilt (*t*-test *p* = 0.2), and it was significantly different from zero (*t*-test *p* = 0.007). The ocular torsion also drifted in some subjects. This drift was always towards the direction of the head tilt. The average drift for the ocular torsion was −0.8 ± 0.3° during 500 trials (~15 min) with the left head tilt and +0.6 ± 0.2° with the right head tilt. The drift of ocular torsion was also significantly different from zero (*t*-test *p* = 0.001) and symmetrical (*t*-test *p* = 0.5).

Once the head returned to upright position, there was an aftereffect with a significant difference in SVV compared with the baseline in upright position before the head was tilted (*p* = 0.001). The average SVV aftereffect among subjects was −3.9 ± 0.6° following the left head tilt, which was significantly different from the average SVV aftereffect of +2.55 ± 1.0° following the right head tilt (*t*-test *p* = 0.00002). These aftereffects were however symmetrical after reversing the direction of individual aftereffects for one head tilt position (*t*-test *p* = 0.3). The average aftereffect in the ocular torsion was +0.2 ± 0.3° after the right head tilt and −0.2 ± 0.3° after the left head tilt. There was no significant difference between torsion aftereffects with right and left head tilt (*p* = 0.3; Figure 2B).

**Figure 3:**
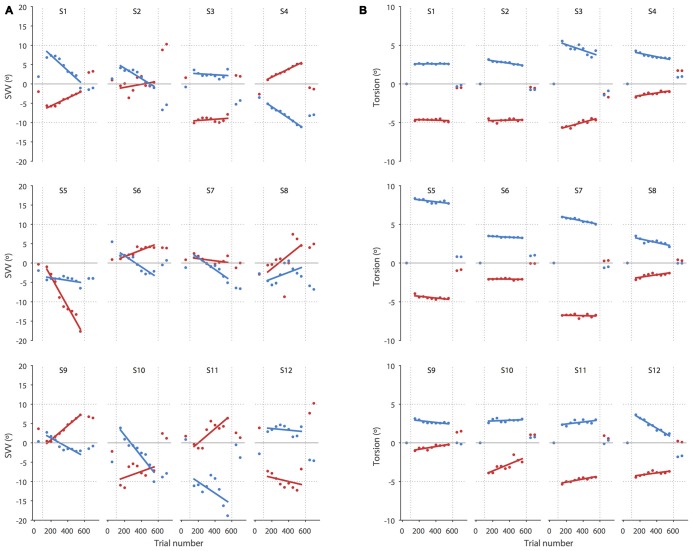
**SVV and torsional position of the eyes during prolonged head tilts are shown in individual subjects. (A)** Each point corresponds with the SVV calculated from 100 trials for the right (red) and left (blue) head tilts. The gaps in the data correspond with the first 50 trials in the new head position where SVV estimates were not reliable and were discarded. **(B)** As in the SVV plot, each point corresponds with the average ocular torsion from blocks of 100 trials for the right (red) and left (blue) head tilts. The gap in the data also corresponds with the first 50 trials that was discarded. Solid lines in both SVV and torsion plots represent linear regressions used to estimate the amount of drift.

**Figure 4:**
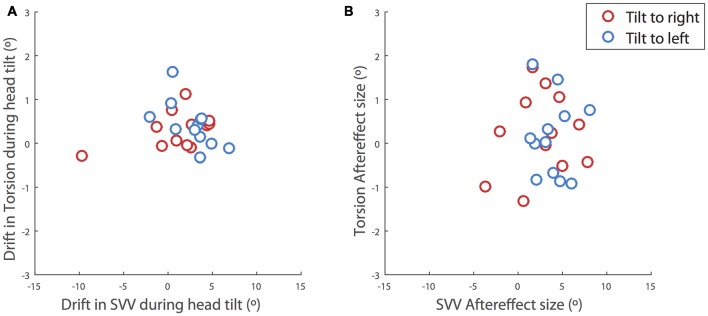
**Correlations between the SVV and ocular torsion. (A)** During head tilt and **(B)** after the head returned to upright position. The drift during head tilt for each subject is estimated as the slope of a linear fit to the SVV responses or ocular torsion. The plots include the values for the right head tilt (red) and the left head tilt (blue). The values for the right head tilt are reversed and shown along with the values for the left head tilt. There is no significant correlation between the drifts of SVV and ocular torsion (*r* = 0.02, *p* = 0.6) or their aftereffects (*r* = 0.2, *p* = 0.6).

Even though the SVV drift and aftereffect were consistently in the same direction, the amount of aftereffect did not show a significant correlation with the amount of drift (*p* = 0.4). That is, SVV drifts to the left or right were usually followed by aftereffects to the left or right respectively, although larger drifts were not necessarily followed by larger aftereffects (Figure 3). The correlation between the drift and aftereffect for ocular torsion was also not significant (*p* = 0.07). Next we looked at the relationship between the drifts in SVV and ocular torsion during head tilt and also their aftereffects when the head returned to upright position. There was no significant correlation between the drifts of SVV and ocular torsion (*r* = 0.02, *p* = 0.6) or their aftereffects (*r* = 0.2, *p* = 0.6; Figure 4).

Our SVV paradigm was partially time constrained and the recording duration depended on the reaction time for individual trials. Thus some subjects finished the experiment faster as they were quicker in their responses. We tested whether there was a relationship between the reaction time and SVV error, SVV drift or SVV aftereffect, but there was no such correlation (*p* > 0.5 in all cases). When SVV and ocular torsion were aligned in time instead of trial numbers, the same patterns of drift were seen during head tilt (Figure 2C). The drift aligned in time was symmetrical, i.e., no difference between the values for the right tilt and the reversed values for the left tilt (*t*-test; SVV *p* = 0.3, torsion *p* = 0.6), and it was significantly different from zero (*t*-test; SVV *p* = 0.01, torsion *p* = 0.003).

We also compared the total duration of head positions (16.1 ± 0.6 min for right head tilts and 15.7 ± 0.3 min for left head tilts) among subjects, which showed no significant difference, either for SVV drift or aftereffect (*p* > 0.05 in all cases). The reaction time was significantly larger during head tilt (686 ± 25 ms) when compared with the baseline in upright position (640 ± 28 ms; *t*-test, *p* = 0.004). There was no significant difference between the reaction times in the baseline (640 ± 28 ms) and post-tilt upright positions (661 ± 30 ms, *t*-test, *p* = 0.2). Moreover, there was no significant drift in the reaction time during the head tilts (*p* = 0.7).

We also measured SVV precision with a metric defined as the the difference between two points on the psychometric curve. These points correspond with 50% (chance level) and 75% correct responses for reporting the right tilt during the SVV task. We did not find a significant drift in precision during the head tilts (*p* = 0.4). The precision was worse (2.0 ± 0.3°) while the head was tilted when compared with the baseline in upright position (0.7 ± 0.1°; *t*-test, *p* < 0.001). It was also worse when the head returned to upright position (1.3 ± 0.1°) when compared with the baseline in upright position (*t*-test, *p* < 0.001). We also tested if there was a relationship between the SVV precision and SVV error, SVV drift or SVV aftereffect but there was no significant correlation (*p* > 0.5 in all cases). These results suggest that the drift in SVV responses was not related to the lack of attention or fatigue.

## Discussion

### SVV Drift and Ocular Torsion

Our results show that perception of upright may drift over time during a static head tilt, reflected by a significant change in SVV responses in the direction of the head tilt. This finding is consistent with previous reports of SVV drift during head tilt (Wade, 1970; Tarnutzer et al., 2013; Kheradmand et al., 2015). Ocular torsion is a source of error in upright perception as the orientation of the images on the retina changes with ocular counterroll (Wade and Curthoys, 1997; Pavlou et al., 2003; De Vrijer et al., 2009). Here we addressed a possible link between the drift in upright perception and torsional eye position by tracking SVV responses and ocular torsion simultaneously. We found no significant correlation between the drifts in upright perception and ocular torsion; that is, subjects with larger drift in ocular torsion did not necessarily have larger drifts in SVV responses. In addition, while the average torsion drift was in the same direction as the SVV drift for the left and right head tilts, the drift in torsion was smaller and more consistent across subjects than the SVV drift, which varied from subject to subject (Figure 3). Therefore, the torsional eye position—or its driving input from the otoliths—may not be the source of drift in upright perception during head tilt. A similar drift has been shown when upright perception was measured by a haptic task, also suggesting that the drift cannot be entirely related to visual errors induced by ocular torsion (Wade and Day, 1968a; Tarnutzer et al., 2013).

### SVV Aftereffect and Ocular Torsion

We found a significant SVV bias in the tilt direction after the head returned to upright position. There was however no significant aftereffect in ocular torsion following head tilt. Others studies have also shown aftereffect in upright perception using either visual (i.e., SVV) or haptic tasks, however ocular torsion has not been measured previously (Wade and Day, 1968a,b; Tarnutzer et al., 2013). With the visual task, the magnitude and direction of aftereffect were not different in the supine and upright body positions, suggesting that neck proprioception was the primary source of adaption in upright responses during head tilt (Day and Wade, 1968).

We did not find a significant correlation between the drift in SVV responses and aftereffect consistent with findings from Tarnutzer et al. (2013). However, while most of their subjects showed a drift in SVV responses during head tilt, there was no significant drift in the group data due to variability among individual subjects. In addition, some subjects showed aftereffects in the opposite direction of the drift while in our results the SVV aftereffect and drift were consistently in the same direction. The difference in these findings could be related to how the head was tilted in each study. In our experiment the head was tilted on the body whereas in Tarnutzer et al. (2013) the head and body were tilted en bloc. As previously reported, SVV aftereffect with head tilt alone could be larger than the aftereffect with whole-body tilt (Wade, 1970). Also, when SVV was measured in different head and body tilt positions, the error was aligned with the head axis as the reference frame, and not the trunk axis (Guerraz et al., 1998; Tarnutzer et al., 2010). These findings together suggest that the adaptive changes in upright perception are mainly related to the head position relative to the body, and not the trunk or head position relative to gravity.

### SVV Drift and Aftereffect Explained by the Bayesian Spatial-Perception Model

An intuitive and effective computational model for static upright perception in the dark is based on the Bayesian approach (MacNeilage et al., 2007; De Vrijer et al., 2009; Clemens et al., 2011). In this framework, the upright estimate is determined by a weighted average of existing knowledge (prior) and noisy sensory information. Here we examined whether the drift and aftereffect pattern in SVV responses could be simulated by changes in individual parameters of the Bayesian model (De Vrijer et al., 2009).

The SVV error in the Bayesian spatial-perception model is determined by the head-in-space and eye-in-space estimates:

(1) $$\begin{array}{l} {\text{μ}}_{\text{SVV}}\;=\;\left({\text{H}}_{\text{S}}-{\text{μ}}_{\tilde{{\text{H}}_{\text{S}}}}\right)+\left({\text{E}}_{\text{H}}-{\text{μ}}_{\tilde{{\text{E}}_{\text{H}}}}\right)\;\\ \;=\;\frac{\sigma_{\hat{{\text{H}}_{\text{S}}}}^{2}}{\sigma_{{\text{H}}_{\text{Sp}}}^{2}+\sigma_{\hat{{\text{H}}_{\text{S}}}}^{2}}\cdot {\text{H}}_{\text{S}}-\Delta {\text{E}}_{\text{H}}\sin\left({\text{H}}_{\text{s}}\right) \end{array}$$

In Equation (1) μ_svv_ represents SVV error, H_s_ actual head-in-space position, ${\text{μ}}_{\tilde{{\text{H}}_{\text{S}}}}$ estimated head-in-space position, E_H_ actual eye-in-head position, and ${\text{μ}}_{\tilde{{\text{E}}_{\text{H}}}}$ estimated eye-in-head position. As a convention, all the sensory inputs are denoted by the hat symbol (∧) and all the estimates obtained by sensory integration are denoted by the tilde symbol (~). For example, $\hat{{\text{H}}_{\text{S}}}$ represents the head orientation in space as measured by the head-in-space sensors, and $\tilde{{\text{H}}_{\text{S}}}$ represents the final head-in-space estimate by the brain. Among the sensory inputs to the model, the head-in-space input ($\hat{{\text{H}}_{\text{S}}}$) is noisy but unbiased by systematic errors (with a variance of $\sigma_{\hat{{\text{H}}_{\text{S}}}}^{2}$), and the prior (with variance of $\sigma_{{\text{H}}_{\text{Sp}}}^{2}$) is taken into account to estimate head position (${\text{μ}}_{\tilde{{\text{H}}_{\text{S}}}}$). Since we spend most of our time in upright position, the prior for head position is a Gaussian distribution that peaks at zero (i.e., upright position). This results in an underestimation of upright at larger head tilt angles as the weighted estimate of head position is biased by the prior (i.e., A effect). De Vrijer et al. (2009) added a free parameter to the model (ΔE_H_) that accounts for an error in estimating ocular torsion by the brain. This “uncompensated” ocular torsion can explain the SVV error in the opposite direction at smaller head tilt angles (i.e., E effect). The model also allows for a lower precision of the head-in-space inputs ($\sigma_{\hat{{\text{H}}_{\text{S}}}}^{2}$) at larger head tilt angles. This is formulated by

(2) $$\sigma_{{\hat{\text{H}}}_{\text{S}}}\;=\;{\text{α}}_{0}+\;{\text{α}}_{1}\left|{\text{H}}_{\text{S}}\right|$$

in which α_0_ represents the noise in upright position and α_1_ accounts for the proportional increase of noise with head tilt. Note that this model assumes a vertical orientation of the trunk. Thus, the sensed head orientation in space ($\hat{{\text{H}}_{\text{S}}}$) is a combination of the otolith and proprioception signals.

**Figure 5:**
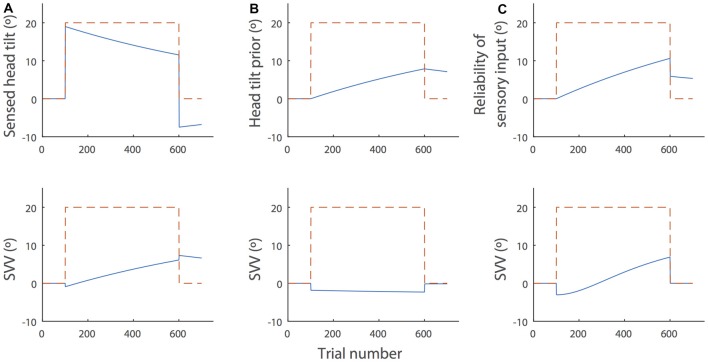
**Simulations of the SVV drift and aftereffect during and after prolonged head tilt (dashed red lines) by the Bayesian spatial-perception model.** The top panels show changes in the individual parameters of the model over time and the bottom panels show the SVV drift produced by the model in each scenario (blue lines). For these sample simulations, we used the parameter values from subject SR in Table 2, De Vrijer et al. (2009). **(A)** Head-in-space sensory input ($\hat{{\text{H}}_{\text{S}}}$): sensory inputs typically decrease over time in the presence of a constant stimulus and there is often an opposite response after the stimulus is removed. In this context, the decay in head-in-space sensory inputs during head tilt would result in the drift of SVV in the direction of the head tilt. After the head returns to upright position, the opposite sensory response would cause the aftereffect. This scenario is consistent with the actual SVV drift and aftereffect in our data. **(B)** The head position prior (H_Sp_): the prior might gradually drift towards the actual head position during head tilt. In this case, however, the SVV drift from the model would be in the opposite direction of the head tilt and it does not match the actual pattern of SVV drift. **(C)** Increase of the sensory noise (α_1_): the head-in-space sensory estimate may become less reliable over time (i.e., noisier), which would gradually increase the weight of the head prior in SVV responses. This scenario also produces an SVV drift toward the actual head position. However, it could not account for the SVV aftereffect, because when the head returns to upright, the noise will reduce to the baseline (α_0_), irrespective of the change in the value of α_1_ (see Equation 2).

We used individual fit parameters of the Bayesian model to simulate the pattern of SVV drift and aftereffect. These parameters included the prior for head position (H_Sp_), the head-in-space sensory input ($\hat{{\text{H}}_{\text{S}}}$) and the sensory noise with tilt angle (α_1_; Figure 5). Since the model does not allow for large changes in SVV from small changes in ocular torsion—see Equation (1)—and because we did not observe a large drift in ocular torsion, ΔE_H_ was not included as a possible source of SVV drift. Equation (1) was also modified to allow the head position prior (H_Sp_) to drift as a function of time with values different from zero:

(3) $${\text{μ}}_{\text{SVV}}\;=\;\frac{\sigma_{{\text{H}}_{\text{Sp}}}^{2}}{\sigma_{{\text{H}}_{\text{Sp}}}^{2}+\sigma_{\hat{{\text{H}}_{\text{S}}}}^{2}}\cdot {\text{H}}_{\text{S}}-\frac{\sigma_{\hat{{\text{H}}_{\text{S}}}}^{2}}{\sigma_{{\text{H}}_{\text{Sp}}}^{2}+\sigma_{\hat{{\text{H}}_{\text{S}}}}^{2}}\cdot {\text{H}}_{\text{Sp}}-\Delta {\text{E}}_{\text{H}}\text{sin}\left({\text{H}}_{\text{s}}\right)$$

Among all the fit parameters, only the drift in head-in-space sensory inputs ($\hat{{\text{H}}_{\text{S}}}$) could produce the pattern of SVV drift and aftereffect in our data (Figure 5A). The head-in-space information is a combination of the otolith and proprioceptive inputs. Since the changes in ocular torsion during head tilt were much smaller than the changes in SVV and there was no significant correlation between the drifts of SVV and ocular torsion, the proprioceptive inputs—and not the otoliths—could be the main source of the drift in the head-in-space sensory input ($\hat{{\text{H}}_{\text{S}}}$). This finding is line with previous studies that suggest neck proprioception is the source of SVV drift and aftereffect (Wade and Day, 1968a; Wade, 1970). Other simulation scenarios included the drift of head prior (H_Sp_) toward actual tilt position and the increase in sensory noise (α_1_) during head tilt. In case of the drift in H_Sp_, we examined whether a shift of the prior toward the head tilt position could explain the drift in upright perception. In such case, however, the SVV would drift in the opposite—and not toward—the direction of the head tilt (Figure 5B). In case of the increase in α_1_, we examined whether the drift in upright perception could be related to an increase in sensory noise during head tilt. In this case, $\hat{{\text{H}}_{\text{S}}}$ becomes less reliable over time during head tilt, which gradually increases the weight of the head prior in SVV responses. This scenario also produces an SVV drift toward the actual head position. However, when the head returns to upright, the sensory noise would reduce back to the baseline (α_0_) and thus it does not result in an aftereffect (Figure 5C). While the drift in head sensory inputs (Figure 5A) can explain our result, it does not account for a lack of correlation between the magnitudes of SVV drift and aftereffect. Thus, it is possible that other scenarios above (Figures 5B,C) also affect the SVV drift and cause variability among individuals while the aftereffect is not influenced. Future studies will have to consider more sophisticated state-space models such as Kalman filters to address the possibility of SVV drift based on the internal estimates of the model and not just its parameters.

In sum, our results show that SVV drift during head tilt and its corresponding aftereffect in upright position were not correlated with changes in ocular torsion. These results along with simulations from the Bayesian spatial-perception model suggest that proprioception could be the source of drift in upright perception during head tilt, and subsequently the aftereffect in upright position. To verify this hypothesis, SVV drift and ocular torsion could be measured under different combinations of whole-body and head-on-body tilts. For example, we expect to find smaller drifts in upright perception during whole-body tilts. To estimate head orientation from neck proprioception, the brain must also estimate body orientation (Clemens et al., 2011; Fraser et al., 2015). Future experiments could dissociate the effects of neck and body proprioception and also test if the results are consistent for small and large tilt angles.

## Author Contributions

JO-M and AK contributed to all aspects of this study including the conception and design of the experiments and acquisition, analysis and interpretation of the data.

## Funding

This work was supported by grants from the National Institute of Deafness and Other Communication Disorders (NIDCD); 5K23DC013552, and the Leon Levy and Fight for Sight foundations.

## Conflict of Interest Statement

The authors declare that the research was conducted in the absence of any commercial or financial relationships that could be construed as a potential conflict of interest.
